# Identification of Emotional Facial Expressions: Effects of Expression, Intensity, and Sex on Eye Gaze

**DOI:** 10.1371/journal.pone.0168307

**Published:** 2016-12-12

**Authors:** Laura Jean Wells, Steven Mark Gillespie, Pia Rotshtein

**Affiliations:** School of Psychology, University of Birmingham, Birmingham, United Kingdom; Vanderbilt University, UNITED STATES

## Abstract

The identification of emotional expressions is vital for social interaction, and can be affected by various factors, including the expressed emotion, the intensity of the expression, the sex of the face, and the gender of the observer. This study investigates how these factors affect the speed and accuracy of expression recognition, as well as dwell time on the two most significant areas of the face: the eyes and the mouth. Participants were asked to identify expressions from female and male faces displaying six expressions (anger, disgust, fear, happiness, sadness, and surprise), each with three levels of intensity (low, moderate, and normal). Overall, responses were fastest and most accurate for happy expressions, but slowest and least accurate for fearful expressions. More intense expressions were also classified most accurately. Reaction time showed a different pattern, with slowest response times recorded for expressions of moderate intensity. Overall, responses were slowest, but also most accurate, for female faces. Relative to male observers, women showed greater accuracy and speed when recognizing female expressions. Dwell time analyses revealed that attention to the eyes was about three times greater than on the mouth, with fearful eyes in particular attracting longer dwell times. The mouth region was attended to the most for fearful, angry, and disgusted expressions and least for surprise. These results extend upon previous findings to show important effects of expression, emotion intensity, and sex on expression recognition and gaze behaviour, and may have implications for understanding the ways in which emotion recognition abilities break down.

## Introduction

Accurate identification of emotional facial expressions (EFEs) is essential for everyday social interaction. However, the extent to which EFEs are generated for the purpose of social interaction, or are byproducts of the emotional experience, has been subject to some debate [1–2]. The importance of communicating EFE information is emphasized by results showing that the processing of human EFEs is optimized [3–4], and that the processing of certain EFEs occurs even when the face is presented outside of conscious awareness [5–6]. Despite these findings, it has been argued that the processing of emotional faces nonetheless requires top-down control of attention [7].

Attentional allocation for emotional faces may be measured through the use of eye tracking techniques, with a close relationship observed between eye movements and spatial attention [8–9]. Using these techniques, Eisenbarth and Alpers [10] showed that the recognition of human EFEs is dependent upon information from two main areas of interest (AOI): the eye region and the mouth region. However, the recognition of emotional expressions varies in relation to factors such as (a) the emotional face and (b) the characteristics of the observer. For example, the processing of human EFEs depends on the emotional content of the expression, with differences in accuracy and response times for different expressions previously reported [11]. Furthermore, the relative importance of diagnostic information obtained from the eye and the mouth regions depends on the expressed emotion [10, 12]. In this study, we revisited the topic of recognizing EFEs in an attempt to systematically assess the impact of various factors on accuracy, response times, and attention allocation to different features. Specifically, we focused on four factors that may affect the processing and classification of EFEs: the type of expression, the intensity of the expression, the sex of the face, and the gender of the observer.

### Type of Expression

Earlier studies [13–17], as well as more recent ones [18–20], provide evidence for six basic facial emotional expressions, referred to as anger, disgust, fear, happy, sad, and surprise. These emotions can be successfully differentiated between and are identifiable cross culturally at an above chance level [18, 21]. Furthermore, research has consistently shown that the emotional content, or type of emotion expressed, can affect the accuracy with which EFEs are recognized [11, 22, 23]. It is also argued that humans are biologically “hard-wired” to recognize threat [24], such as that conveyed by a fearful or angry facial expression, and this position is supported by more rapid detection of angry facial expressions compared to happy expressions when situated amongst neutral stimuli [25].

Surprisingly however, this threat recognition advantage does not translate to accuracy in the explicit recognition of EFEs. Rather, it is reported that happiness is the most accurately and rapidly recognized EFE, an effect known as the ‘happy face advantage’ [11, 22, 23], while fear is the least accurately recognized [11]. This suggests that different expressions may vary with respect to their function: while smiles may be aimed primarily at social communication, fearful and other threat related EFEs may represent the byproduct of an emotional experience. Here, threat related EFEs may be used as a cue to indicate potential danger in the environment (e.g., to indicate the presence of predator) and this can be acted upon by observers. Importantly, the communication of danger by threat related EFEs may be facilitated in the absence of explicit awareness or identification [26–27].

The eye region and the surrounding area represents the most diagnostic facial feature for accurate EFE identification. The importance of the eye region has been demonstrated using a partial masking method (bubbles) to show that information from the eyes (including the eyebrows) is key for the accurate recognition of all expressions [12]. This effect was primarily evident for recognition by a computer model, but also for human observers (though not for the surprised expression). This work also suggests that different features vary in the way that they contribute to the recognition of the different expressions. Where information from the eyes contributes to fear, anger and sad recognition, information from the mouth contributes to the recognition of happy, surprise and disgust. These findings resonate with earlier results showing that fear, anger and sad are better recognized based on the top half of the face; while happiness, disgust and surprise are better recognized from the bottom half [28].

Results from eye tracking studies also support these observations. These studies have typically focused on the eye region and the mouth region as two main areas of interest (AOI), and have shown that dwell time is typically greater on the eye region across different types of expression [10]. It was also shown the dwell time on the eyes was about ~35% longer than on the mouth for sad, fear, anger and neutral expressions, while for happy it was just 25% longer. Greater attention to the mouth of happy compared with other expressions suggests that the mouth may play a more important role in identifying happy expressions. Conversely, the importance of scanning the eyes for fear recognition has been demonstrated by many studies [29–31]. For example, Adolphs et al. [29] found that SM, a patient who showed impaired fearful face recognition, also made fewer spontaneous saccades toward the eye region relative to healthy controls. However, SMs fear recognition recovered when she was instructed to look at the eye region [29].

### Intensity of Expression

Everyday expressions are typically displayed with low to mid intensities [32], and as such, expressions of varying intensity may provide more life-like representations [33]. Varying the intensity of emotional expressions can also make emotion recognition tasks more sensitive to subtle differences in the processing of different EFEs [34]. Although there have been fewer studies investigating the effects of expression intensity compared to the type of expression, the consensus is that when the intensity of an EFE increases, the accuracy of identification also increases [35–36]. This suggests that individuals are, in general, less accurate at identifying more subtle expressions and more accurate when EFEs are more intense. However, advantages for more intense expressions might reflect that recognition is often measured using forced choice responses where the options do not include neutral [35–36]. Thus, it is possible that such methodological designs artificially force participants to attribute an emotion to a face that they would normally perceive as non-expressive.

Intensity has different effects on expressions [37], although these effects do not appear to be consistent. Hoffmann and colleagues [37] examined accuracy for expressions at 50% and 100% intensity, and report that changes in intensity had no effect on emotion recognition for fear and surprise expressions [37]. However, using a different sample of participants the same study reported that changing expression intensity had particular effects for expressions of anger, fear, and sadness. A different study suggests that recognition of happy, and to a lesser degree sad and disgust expressions, follows a sigmoidal shape in which performances asymptote after 60% intensity [35]. Thus, despite some inconsistencies in the effects reported, the impact of intensity on EFE identification may differ according to the emotional content of the expression.

If the primary function of EFEs is aimed at social communication, then a similar pattern of results would be expected for response times and dwell time as has been observed for recognition accuracy. That is, the more ambivalent the expression then the slower the response time, and the more participants will scan the face for additional information. Indeed, Guo [38] showed an inverse relationship between fixation count and expression intensity (20%-100%), with more fixations on lower intensity expressions. However, the effect reached an asymptote after 60% intensities. The increase was observed for both the eyes and the mouth, and so the relative contribution of each feature to emotion identification was unaffected by expression intensity.

### Sex of the face displaying the expression

As well as expression type and intensity, the sex of the face can also affect the identification of EFEs. In general, one of the most common beliefs across cultures with regards to gender and emotion is that women are more “emotional”, with women being expected to experience and express emotions more than men [39]. In line with this, studies have shown that women are typically more facially expressive than men [40], and that females’ non-verbal cues are more accurately judged [41]. The expectation therefore may be that all expressions are judged with more accuracy from women’s than from men’s faces. However, a range of research has demonstrated that the effects of sex may vary with the type of expression [35, 42].

Two complementary theories have been proposed to describe the relation between face gender and expression. The stereotype theory of emotion recognition suggests that a division exists between masculine emotions and feminine emotions [40, 43]. Specifically, anger and disgust are culturally viewed as more masculine and are associated with power; while happiness, sadness, and fear are culturally classed as more feminine and are less associated with power [39, 44]. Theoretically, if expressions are primarily aimed at social communication, it is expected that such stereotypical beliefs will affect recognition accuracy. A ‘Structural Similarities’ explanation suggests that the link between sex and emotions is not culturally driven but is based on the morphology of emotional facial expressions. Thus, sex related differences in face shape are associated with differences in expressive features. Zebrowitz and colleagues [45] support this idea by demonstrating gender specific objective similarities between the appearances of certain emotional expressions using a connectionist modelling approach. They found that neutral male facial expressions showed greater similarity to angry expressions than did female faces, while neutral female faces showed greater similarity to surprise faces [45].

An advantage for recognizing happy expressions from female faces has been repeatedly reported [35, 42, 46]. It has also been shown, albeit with less consistency, that disgust [35] and anger [46] are recognized better from male faces. Nonetheless, not all evidence is consistent with the stereotype or structural similarities theories. For example, Hess et. al. [35] showed that sadness was better recognized from male faces, while Tucker and Friedman [47] found that angry female faces were more accurately judged than sad female faces.

### Gender of the observer

The gender of the person identifying the emotion is a further variable of interest that may affect eye scan paths and recognition of EFEs. Like the belief that women are more emotionally expressive, it is also assumed that women are superior to men at recognizing facial expressions of emotion [48–49]. The primary caretaker theory [50] attempts to explain this notion using evolutionary theories attributing human expression recognition superiority to females’ role in caring for offspring. Specifically, a mother who is more attuned to the emotions of her infant is more likely to promote a secure attachment, which in turn may lay the foundations for healthy development and functioning [48]. Similarly, it is also hypothesized that woman have higher empathizing capacity [51], which again may provide advantages when attempting to read the expressions of others [52].

Currently available evidence regarding female superiority in judging facial expressions is mixed. Montagne and colleagues [49] demonstrated an overall female superiority in a task measuring the processing of emotional faces. However, a meta-analysis revealed that out of 55 studies, only 11 showed a reliable female advantage in EFE recognition abilities [53]. It has been argued that female superiority might only be revealed when the amount of visual information is limited, either by manipulating the exposure duration [48] Hampson et. al., 2006), or the intensity of the expressions [37, 49, 54].

However, others have either found a limited effect of the sex of the observer on EFE recognition [55–56], even under limited exposure durations [57], or did not report an interaction of observer sex with expression intensity [52]. When considering different outcome measures used, the female superiority effect appears to be more reliably associated with differences in response time than with differences in accuracy [48, 56, 58].

The fitness to threat hypothesis predicts that a female superiority effect exists only for negative EFEs, including fear, disgust, sadness, and anger. This is due to the likelihood that negative emotions signal a potential threat to the infant [48]. However, again the evidence in support of this theory is mixed and inconsistent. Hampson et. al. [48] found evidence for a female superiority effect in response times for the recognition of negative emotions in particular. In contrast, others have found that men outperform women when identifying anger, but only when judging the emotion from other male faces [59].

In relation to eye scan paths for EFEs, it is suggested that although both male and female participants show a preference for the eye region, females typically attend more to the eye region compared with male participants, while male participants show greater attention to the mouth than do females [58].

### The Present Study

In the current study we revisited the question related to different factors affecting the classification of EFEs. We specifically focused on four factors: expression type, expression intensity, face sex, and observer sex. We tested both men and women participants, measuring accuracy and reaction time (RT) for the identification of emotional expressions varying in expression, intensity and sex. We also recorded dwell-time (i.e. the total duration of eye gaze) on the two key areas of interest (AOIs): the eyes and the mouth. We note that our AOIs were relatively large, with the mouth including the philtrum or Cupid’s bow area (bottom of nose) and the eyes included the eyebrows and the naison point (top of the nose). It has been suggested that the two latter regions are crucial for recognizing disgust and anger, respectively. We asked participants to identify EFEs from both male and female faces, showing expressions of the six core emotions, at varying levels of intensity. We manipulated intensity using morphs from neutral to a full-blown expression, and expressions were presented at 10%, 55% and 90% intensity. Participants made forced choice responses from seven options: angry, disgust, fear, happy, sad, surprise, and neutral. Although none of the faces presented a fully neutral expression, this option was included to examine the extent to which low intensity expressions are perceived as neutral or are correctly judged to show emotional content. The inclusion of a neutral option represents one attempt to eliminate methodological limitations surrounding the use of forced choice designs that do not allow the participants to label expressions as showing no emotional content.

The investigation of a variety of factors in this study (expression type, expression intensity, face sex, and observer sex) allows for a more in depth examination of those factors that contribute to EFE recognition and how these may interact with one another. Previous studies have generally opted to investigate a minimal number of variables that affect EFE recognition at any one time. This may account for some discrepancies in the literature, including contradictory findings around the impact of observer gender [49, 53]. Some have found a general female superiority effect, while others have found female superiority for only more subtle expressions [37, 49, 54]. Further, the use of multiple outcomes, including accuracy, RT, and dwell time allows for a more comprehensive understanding of the impact of these differing factors.

### Hypotheses

Expression type: we predicted that happy faces would be identified the quickest and most accurately consistent with robust evidence for a ‘happy face advantage’, while fearful expressions would be least accurately identified. We predicted that in general dwell time would be greatest on the eyes compared to the mouth. However, we predicted that dwell time would be relatively higher on the mouth of happy expressions, and on the eyes of fearful expressions.

Expression Intensity: We expected an increase in accuracy, and a reduction in RT and dwell-time, with increasing intensity. We expected that these differences would asymptote earlier for happy faces, with smaller differences in accuracy, RT, and dwell time between 55% and 90% expressive happy faces compared with other expression types.

The sex of the face: we predicted that happy expressions would be recognized best from female faces. However, evidence is mixed with respect to the effects of sex on recognizing other expressions. Theoretically (see above), it was hypothesized that sad and fear would be better recognized from female faces, while anger and disgust would be better recognized from male faces. Previous studies did not suggest different scanning patterns for male and female faces [52, 54].

The observer gender: We predicted that relative to male participants, female participants would respond faster in all conditions. We also predicted that there would be a female superiority effect in accuracy for low intensity expressions in particular. Finally, we also predicted that dwell times on the eyes would be longer among female compared with male participants.

## Method

### Participants

We recruited 39 participants (20 female; 19 male) from the undergraduate student population of a UK based University. Participants ranged in age from 18 to 27 years (*M* = 20.36, *SD* = 1.91). The ethnicities of the participants were white Caucasian (n = 31) and Asian (n = 5). Some participants reported their ethnicity as ‘mixed’ (n = 2) and one participant chose not to report this information. All participants grew up in the UK. Participants were recruited through either the University of Birmingham research participation scheme, in return for course credit, or through volunteering in return for payment of £6.00. The University of Birmingham Committee for Ethical Review for Science, Technology, Engineering, and Mathematics granted ethical permission for this study. Each participant provided his or her written informed consent before the study began.

### Materials

The EFE stimuli were chosen from the NimStim Face Stimulus Set ([60]; http://www.macbrain.org/resources.htm). Ten Caucasian models were chosen (5 female, 5 male), each demonstrating seven expressions: the six universal EFEs (happy, sad, angry, afraid, surprise, disgust) and a neutral expression. To ensure the expressions would be recognized reliably, the selection of faces was based on the NimStim norms data for recognizing expressions. The stimuli chosen included faces with open mouths for some of the expressions. The choice of whether to include an open or a closed mouth expression for each model was based on the NimStim validity data, with the most reliably recognized alternative being selected. Of the ten individual models, the number of open mouthed stimuli selected for each expression were as follows: eight angry, nine disgust, nine fear, ten happy, two sad, ten surprise, and seven neutral.

To obtain different intensities of EFEs, each expression was morphed from the neutral face to 100% expressive using the STOIK Morph Man morphing software (http://www.stoik.com/products/video/STOIK-MorphMan/). Three different intensities were selected for each EFE for each model: normal intensity (90%), moderate intensity (55%), and mild intensity (10%). This gives 18 expressions per model, with 180 faces in total. For example stimuli, see Gillespie et. al. [61]. We used an EyeLink 1000 head mounted eye tracking system (SR Research Ltd.) to record eye gaze and dwell time. Although viewing was binocular, only movements of the participant’s left eye were recorded. Gaze location was sampled once every millisecond.

### Procedure

A full factorial design was used with the following within factors: type of expression (anger, disgust, fear, happy, sad, surprise); intensity (10%, 55%, 90%); face sex (female, male); and observer gender (woman, man) as a between subject factor. A total of 360 trials were presented, with 10 trials per condition. We presented each stimulus (specific face with a specific expression and intensity) only twice, in two separate sessions, to minimize familiarization effects. The order of trials within each session was randomized. Each trial started with a fixation point presented for 500ms followed by the presentation of the face. Participants were asked to press the number on the keyboard which corresponds to the answer they think is correct for each face. The seven possible answers (the six emotions and neutral) were listed vertically on the left side of the screen with a corresponding number for each emotion: 0 = neutral, 1 = angry, 2 = disgust, 3 = fear, 4 = happy, 5 = sad, 6 = surprise. Participants were asked to respond as fast and as accurately as possible. There was no time limit on each trial and the next trial would only begin after a response had been made.

At the beginning of the experiment, a calibration and validation procedure was completed using 9 points, one at fixation, and the rest at the edge of the screen. Eye tracking was recalibrated after every 20 trials. Another 9 points calibration occurred after 120 trials.

### Analysis

The analysis focused on three parameters: Accuracy, RT, and Dwell-time. An accurate response was defined as selecting the correct EFE for each trial. ‘Neutral’ responses were counted as inaccurate. The number correct for each emotion, at each level of intensity, for male faces and females faces, varied between 0 and 10. This is based on having five male and five female models showing each expression at each level of intensity, with each unique stimulus presented twice. RT was the time taken to make a response independent of whether that response was correct or incorrect. We measured dwell-time on two predetermined AOIs: the eyes and the mouth. The eye region comprised of a 289x100 pixel rectangle which included both the eyes, the eyebrows and the area in between; the mouth region was a 208x139 pixel rectangle which included the mouth and its surroundings. We measured absolute dwell-time for each AOI, that is, the total amount of dwell-time across all fixations within each AOI.

The data were first analyzed manually. Any trials that had 50% or more of the eye-tracking data outside the face area were deleted. These trials most likely reflect drift in the measurement of eye movements, rather than an accurate representation of a participant’s eye-gaze. The data were then analyzed using a series of ANOVAs and paired *t*-tests. We applied a Bonferroni correction to all *t*-tests. Data for accuracy, RT, and dwell time were first analyzed including the participant’s gender as a between subject factor.

Where we failed to observe predicted effects, we followed this up by computing a Bayes factor (http://www.lifesci.sussex.ac.uk/home/Zoltan_Dienes/inference/Bayes.htm) to assess the strength of the evidence supporting the null hypothesis [62].

## Results

### Accuracy and RT

For analyses of accuracy and RT we used a 2 (face sex: male; female) x3 (intensity: 10%; 55%; 90%) x6 (expression: happy; sad; angry; fear; surprise; disgust) within-subjects ANOVA, with participant’s gender as a between subject factor.

### Effects of participant gender

The main effect of gender of participant showed a significant overall difference in RT *F*(1, 37) = 6.98, *p* < .05, pɳ^2^ = .16, with females overall responding faster than males (see Table 1). Although there was no significant effect of participant gender on accuracy *F*(1, 37) = .06, *p* > .05, pɳ^2^ = .001, a Bayes factor was calculated for this effect given that a clear prediction was made that females would be more accurate than males. This revealed a Bayes factor of 0.19, suggesting that there was evidence in support of the null hypothesis that there was no difference in accuracy between male and female participants.

**Table 1:** Percent correct across intensity and emotion expressed for male and female participants categorizing male and female faces.

		Participant sex (*N* = 39)		
Sex of face	Male (*n* = 19)		Female (*n* = 20)	
		Accuracy *M* (*SE*)		
Male face	28.3 (.56)		27.9 (.44)	
Female face	29.1 (.56)		30.0 (.45)	
		RT *M* (*SE*)		
Male face	2769.99 (163.63)		2225.86 (159.49)	
Female face	2907.44 (168.54)		2236.54 (164.27)	

However, the effect of participant gender on accuracy should be interpreted in light of a significant interaction of participant gender with the sex of the facial stimulus *F*(1,37) = 11.06, *p* < .01, pɳ^2^ = .20 (Table 1). The interaction for accuracy showed that both women and men were more accurate in identifying the expressions of females than males, although the difference was larger for females. The interaction of participant gender with the sex of the face was non-significant for RT *F*(1, 37) = 1.45, *p* > .05, pɳ^2^ = .04.

Although we predicted in particular that there would be a female superiority effect for expressions at lower intensities, the interaction of participant gender with intensity was non-significant for both accuracy *F*(2, 74) = 1.55, *p* > .05, pɳ^2^ = .04, and RT *F*(2, 74) = 1.92, *p* > .05, pɳ^2^ = .05. There were no other effects involving the gender of the participant for either accuracy or RT (*p* > .1).

**Fig 1:**
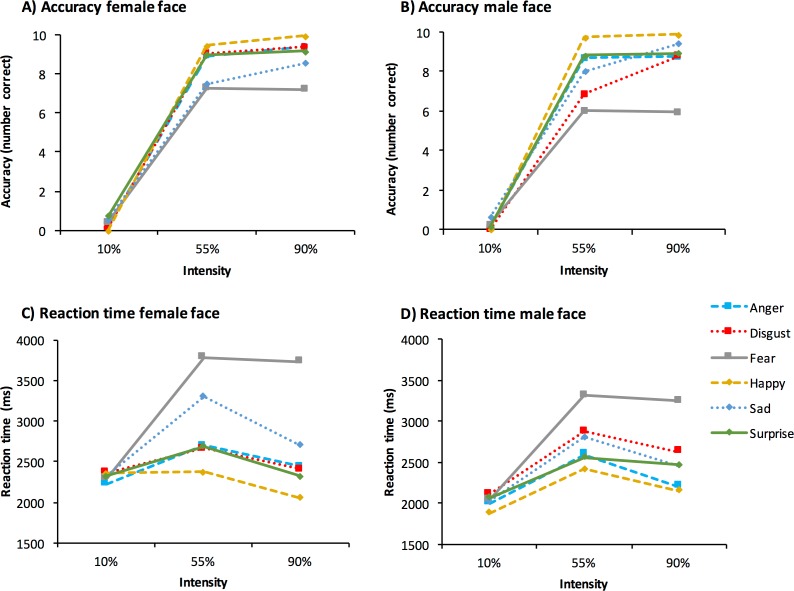
Accuracy of emotion recognition for female (A) and male (B) faces, and response times for classifying female (C) and male (D) faces, by expression, and intensity.

As the gender of the participant did not interact with any of the stimulus related factors, we collapsed across the responses of male and female participants for all subsequent analyses. The results are presented in Fig 1.

### Effects of Expression Type

We observed a main effect of expression type for both accuracy, *F*(5, 190) = 40.69, *p* < .001, pɳ^2^ = .52, and RT *F*(5, 190) = 26.01, *p* < .001, pɳ^2^ = .41. For accuracy, Bonferroni corrected pairwise comparisons showed that fear was the least accurately recognized expression, while happy was judged the most accurately, compared with all other expressions (*p* < .05). Similarly, comparisons for RT showed that fear was recognized the slowest compared with all other expressions, while happy was recognized more quickly than disgust, fear, sad, and surprise (*p* < .05). However, the effects of expression type should be interpreted in light of a significant interaction of expression, intensity, and sex of the face, for both accuracy and RT, described below.

### Effects of expression intensity

**Fig 2:**
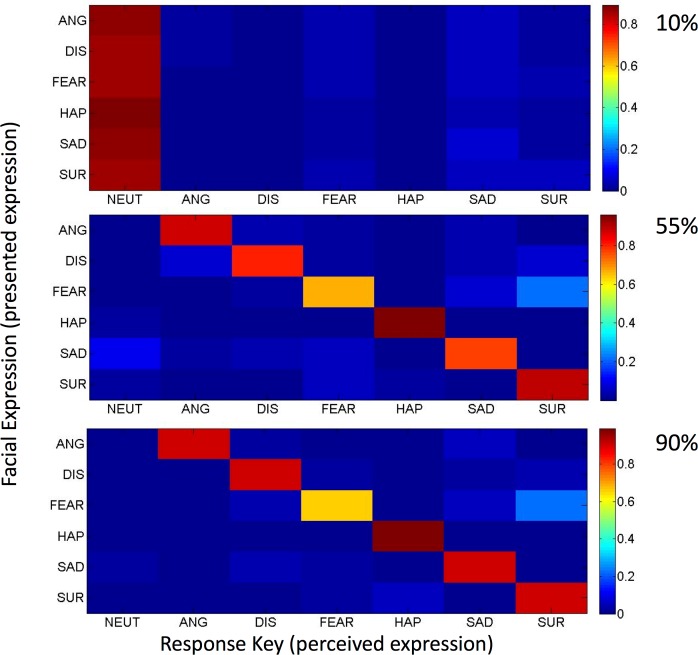
Confusion matrixes showing the percentage of participants’ responses to all six emotions for 10% (A), 55% (B) and 90% (C) intensities.

We also found a significant effect of expression intensity on accuracy *F*(2, 76) = 4961.35, *p* < .001, pɳ^2^ = .99, with higher intensity expressions (10% < 55%, 55% < 90%) associated with a greater degree of accuracy across all emotions (all comparisons *p* < .001). In addition, we found that most (around 80%) of the 10% expressions were categorized as neutral across all expressions (~1.7% std across participants) (see Fig 2). This made for generally low levels of accuracy when judging the lowest intensity (10%) expressions, as neutral responses were classified as incorrect. Hence, overall accuracy appears low in Table 1 given the low number of correctly categorized expressions at 10% intensity.

There was also a significant effect of intensity on RT *F*(2, 76) = 30.86, *p* < .001, pɳ^2^ = .45. However, comparisons showed that the effects were not linear. RTs were slowest for 55% expressions compared with 10% and 90% (*p* < .001), while 90% expressions were judged slower than 10% (*p* < .01). However, the effects of expression intensity should be interpreted in light of a significant interaction of expression, intensity, and sex of the face, for both accuracy and RT, described below.

### Effects of face sex

We also found that although female expressions were judged more accurately than male expressions *F*(1, 38) = 66.33, *p* < .001, pɳ^2^ = .64, male expressions were judged more quickly *F*(1, 38) = 23.44, *p* < .001, pɳ^2^ = .38. However, the observed effects for the sex of the face should be interpreted in light of the below interactions.

### Interactions of expression, intensity and face sex

We found a significant three-way interaction of expression, intensity, and sex of the face for accuracy *F*(10, 380) = 9.78, *p* < .001, pɳ^2^ = >.05. To unpack the interaction we computed separate 2 (sex) x 3 (intensity) ANOVAs for each expression. To account for multiple comparisons we applied a Bonferroni correction, with results interpreted as significant at an alpha level of *p* < .008 (i.e. .05/6). Using this stringent criterion, an interaction of face sex with emotion intensity was observed for disgust and happy expressions (see Table 2). The interaction for fearful and sad expressions did not survive the correction, while interactions for anger and surprise failed to reach significance.

**Table 2:** Simple effects for interaction of face sex with emotion intensity for accuracy and RT for expressions of anger, disgust, fear, happy, sad, surprise.

Expression		Sex x intensity interaction *F* (*p*)	Simple effect pattern (*p* < .05)
Anger	Accuracy	2.20 (*ns*)	
	RT	0.587 (*ns*)	
Disgust	Accuracy	39.80 (< .008)	Female > male at 55%, 90%
	RT	4.47 (< .05)	
Fear	Accuracy	5.12 (< .05)	
	RT	0.67 (*ns*)	
Happy	Accuracy	5.24 (< .008)	Male > female at 55%
	RT	9.68 (< .008)	Female > male at 10%
Sad	Accuracy	3.36 (< .05)	
	RT	0.94 (*ns*)	
Surprise	Accuracy	1.47 (*ns*)	
	RT	3.14 (< .05)	

Note: *ns* = non-significant (*p* > .05).

Results are interpreted as significant using an adjusted alpha level of *p* < .008.

A follow up break down of the disgust and happy expressions for each intensity showed that the sex of the face had no effect for disgust expressions at lower intensity (10%), or for happy expressions at full intensity (90%). Furthermore, an opposite pattern was observed in identifying these two expressions, with participants showing greater accuracy recognizing disgust from female faces, and happy from male expressions. Both of these effects were most pronounced at the 55% intensity (see Table 2). However, the structural similarities and stereotype theories predict better recognition of happy from female faces, and disgust from male faces. We therefore computed Bayes factors to test the strength of the evidence for the null hypothesis. Based on the effects observed by [42], we found a Bayes factor of 0.03 for the effect of face gender on accuracy for both disgust, and happy expressions. As such, when considering the effect size in the expected direction, the current data show support for the null hypothesis.

A three way interaction was also observed for RT *F*(10, 380) = 2.51, p < .01, pɳ^2^ = .06. Applying a similar approach to the above, Bonferroni corrected interactions of intensity and sex were observed for happy expressions only. Bonferroni corrected paired samples *t*-tests showed that the sex of the face had no significant effects on RT for expressions at 55% or 90% intensity; however, female happy expressions took longer to identify than male ones at low intensity. See Table 2 for details.

In summary, the pattern of results for accuracy and RT were similar with respect to the expression manipulation, with fearful expressions being the most difficult to recognize and happy being the easiest. However, the sex of the face and the intensity of the expression had different effects on accuracy and RT. Specifically, we showed that accuracy was higher for female faces (with the exception of happy) but responses were also slower. The extent of this interaction depended on the expression and intensity level, and was most pronounced for disgust and happy expressions. Furthermore, while accuracy results were linearly related to the intensity manipulation, RT results showed that response times were slower for moderate and likely more ambivalent intensity expressions.

### Dwell time

#### Effects of participant gender

The gender of the participant did not affect the gaze pattern or interact with any of the factors for analyses of dwell time. For simplicity in reporting the results we therefore removed participant gender as a between subject factor.

#### Effects of area of interest

**Fig 3:**
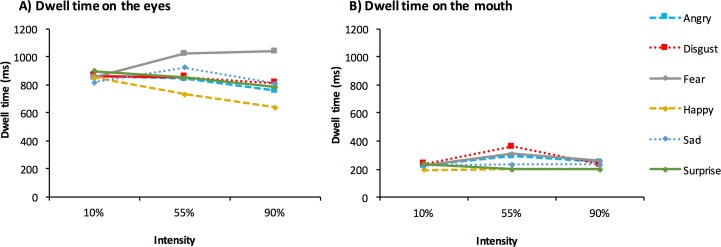
Dwell time on the eyes (A) and the mouth (B) of emotional facial expressions by expression, and intensity.

The results collapsed across the gender of the participant are presented in Fig 3. We used a 2 (AOI: eyes; mouth) x2 (face sex: male; female) x3 (intensity: 10%; 55%; 90%) x6 (expression type: happy; sad; angry; fear; surprise; disgust) within-subjects ANOVA for the analysis of dwell time on the eyes and the mouth. We showed that there was a main effect of AOI *F*(1, 38) = 100.69, *p* < .001, pɳ^2^ = .73, with longer dwell times on the eyes (*M* = 844.49, *SE* = 50.32) compared with the mouth (*M* = 245.53, *SE* = 22.97).

#### Interaction of area if interest with intensity and expression

We also observed a significant interaction of intensity and expression with AOI *F*(10, 380) = 2.36, *p* < .01, pɳ^2^ = .06. To better understand this interaction, we examined dwell times separately for the eye region and the mouth region.

An ANOVA of sex, intensity, and expression for dwell time on the eyes showed a significant interaction of expression and intensity *F*(10, 380) = 9.03, *p* < .001, pɳ^2^ = .19. When further broken down by intensity we observed significant effects of expression at 55% *F*(5, 190) = 9.02, *p* < .001, pɳ^2^ = .19, and 90% intensity *F*(5, 190) = 25.53, *p* < .001, pɳ^2^ = .40. The effect of expression for faces at 10% intensity was non-significant *F*(5, 190) = 1.12, *p* > .05, pɳ^2^ = .03.

Table 3 shows the results of all pairwise comparisons for dwell time on the eyes of 55% and 90% expressions. At 55% intensity, Bonferroni corrected pairwise comparisons (*p* < .003) showed that dwell time on the eyes was greatest for fearful compared to all other expressions except sadness. Dwell time on the eyes of happy expressions was also significantly lower than that for sad and surprised expressions (*p* < .003). At 90% intensity, we again showed that dwell time on the eyes was greater for fearful compared to all other expressions. Furthermore, we also showed that dwell time was shortest on happy eyes compared to all other expressions.

**Table 3:** Paired sample t-tests comparing dwell time on the eye region of emotional expressions at 55% and 90% intensity.

55%	*t*					
90%						
	Angry	Disgust	Fear	Happy	Sad	Surprise
Angry		-.23	-5.32*	1.77	-1.88	-0.15
Disgust	-1.97		-3.60*	3.12	-1.66	.07
Fear	-7.06*	-4.68*		5.45*	2.69	3.76*
Happy	3.87*	5.95*	8.62*		-4.34*	-3.20
Sad	-1.59	.21	5.32*	-7.13*		1.71
Surprise	-.72	.96	5.94*	-6.57*	.93	

Note: top right of table shows comparisons for expressions at 55% intensity, while bottom left shows comparisons for expressions at 90% intensity.

* *p* < .003 (adjusted for multiple comparisons using Bonferroni correction)

The interaction of intensity and expression for dwell time on the mouth was also found to be significant *F*(10, 380) = 3.20, *p <* .001, pɳ^2^ = .08. When broken down by intensity, we showed that there was a significant effect of expression for faces at 55% *F*(5, 190) = 6.31, *p* < .001, pɳ^2^ = .14, and at 90% *F*(5, 190) = 4.60, *p <* .001, pɳ^2^ = .11, intensity. The effect of expression for faces at 10% was non-significant *F*(5, 190) = 1.07, *p* > .05, pɳ^2^ = .03. Fig 3B shows dwell time on the mouth of emotional expressions as a function of the emotion expressed and the intensity of the expression.

Table 4 shows the results of all pairwise comparisons for dwell time on the mouth of 55% and 90% expressions. For faces at 55% intensity, Bonferroni corrected comparisons showed longer dwell time on the mouth of disgusted compared with happy and surprised expressions. Dwell time was also shorter on the mouth of surprised expressions compared with fearful and sad expressions. At 90% intensity, we showed that dwell time was shorter on the mouth of surprised compared with angry and fearful expressions.

**Table 4:** Paired samples t-tests comparing dwell time on the mouth region of different emotional expressions at 55% and 90% emotional intensity.

55%	*t* (*p*)					
90%						
	Angry	Disgust	Fear	Happy	Sad	Surprise
Angry		-2.11	-.75	1.07	.61	2.54
Disgust	1.36		1.74	3.45*	2.72	5.35*
Fear	-.36	-1.28		1.68	1.22	3.68*
Happy	3.03	1.97	2.91		-1.11	2.59
Sad	1.22	.02	1.32	-1.93		3.21*
Surprise	4.07*	2.38	3.65*	-.05	2.05	

Note: top right of table shows comparisons for expressions at 55% intensity, while bottom left shows comparisons for expressions at 90% intensity.

* *p* < .003 (adjusted for multiple comparisons using Bonferroni correction)

## Discussion

Emotional expression recognition represents a crucial part of successful social interaction, allowing one to communicate valence specific information to an observer, and allowing others to infer the emotional state of the expresser. Emotionally salient aspects of the face, namely the eye region and the mouth region, provide diagnostic information for emotion classification [10]. However, accuracy of expression recognition, and attention to the eyes and the mouth, may vary with particular characteristics of the observed expression. Furthermore, there is debate in the literature as to the precise role of EFE information. More specifically, it is debated whether EFEs serve a social interaction function, or are byproducts of the emotional experience. The earlier theory might suggest that different outcome measures should show a similar pattern, with increasing accuracy associated with quicker RTs and shorter dwell times. The aim of this study was to investigate the effects of four factors on emotional expression recognition: expression type, expression intensity, the gender of the face displaying the expression, and the gender of the observer. We start by summarizing the results in relation to our specific hypotheses.

In line with our predictions, happy was the fastest and most accurately recognized expression, and fear was recognized the slowest and least accurately. These findings were in line with our predictions, and are consistent with a ‘happy face advantage’ [12, 22], rather than with a proposed evolutionarily based advantage for judging negative emotions [25]. We also found, consistent with earlier findings [10], that dwell times were longest on the eyes compared to the mouth, and that this increase was largest for fearful expressions. These findings might support the notion that information from the eye region is more salient in the process of recognizing fear compared to other emotions [12, 29], and implies consistency with the suggestion that the widening of the eye whites may represent the critical diagnostic feature for fear [63].

The pattern of results differed for dwell times on the mouth, with longer dwell times for disgust expressions, and shorter dwell times for surprise. Thus, while the eyes may be of relatively reduced importance for recognizing happy expressions, we did not observe relatively increased attention to the mouth. However, when looking only at the earliest fixations on the face, it has been found that more fixations were devoted to the upper lip region of the face for happy/joy expressions compared to the mean [64]. The finding of reduced attention to the eye region for happy faces may reflect the unique shape of the mouth in these expressions, with diagnostic information from the eyes being of relatively less importance [64]. Surprisingly however, when information from the eye or the mouth region is masked, a computer algorithm can still identify happy expressions at above 90% classification accuracy. As such, information from either region should therefore be sufficient for making accurate judgments [65].

It is debated whether the primary role of EFE information is aimed at social interaction, or is a byproduct of emotional experience. The consistent finding of differences in the ease with which different expressions are recognized supports the idea that different expressions serve different primary functions. Smith and Schyns [66] present evidence in favor of differing functions, and show that different EFEs are recognized with varying success over different distances. These authors note that “catastrophic” transformations occur in happy and surprised faces, whereby the mouth opens revealing the teeth. Furthermore, they show that these catastrophic changes are communicated with greater sensitivity over a range of distances, consistent with an explicit function for social interaction for happy and surprised faces. Thus, an explicitly recognizable smiling face might communicate positive emotion and signal that the individual is willing to engage in reciprocal altruism [67].

Conversely, it was found that fear and anger were poorly recognized across a range of viewing distances [66]. As commented by the authors, this finding is surprising for signals communicating potential threat or danger, with the expectation being that such signals should be easily recognizable across a range of distances. Although fear expressions may not serve an explicit social interactional function, these expressions nonetheless serve to communicate a source of threat in the environment. Importantly, this can happen rapidly and in the absence of explicit identification [26–27]. Furthermore, Frith [2] notes that even in the absence of explicit recognition, mimicking the features of a fearful face, that is, widened eyes and dilated nostrils, may also serve to increase vigilance, widening the field of vision and increasing inhalation and sense of smell [68]. Thus, different expressions may diverge in the extent to which their primary function is one of social interaction, or that they reflect a by-product of the emotional experience.

We expected an increase in accuracy and a reduction in RT and dwell time with increasing levels of intensity. In line with our hypothesis, and consistent with the findings of others [35–36], there was an inverse relationship of accuracy with intensity. However, the relationships for RT and dwell time did not follow the expected pattern. Rather, we observed longer RTs for medium intensity, and likely more ambivalent, emotional expressions. That low intensity expressions were categorized the fastest likely reflects that these expressions may have consistently been judged to be neutral. In support of this, the neutral option in the current study was selected in response to 84% of trials displaying a facial stimulus of low (10%) expressive intensity. Thus, participants may have been relatively insensitive to the low levels of emotional content. This pattern of results also suggests that participants found the classification of moderate intensity expressions the most difficult. The findings for modified intensity expressions may most closely resemble the processing of EFEs outside of the lab, with these expressions argued to provide more life-like representations of each expression [33], and to be most sensitive to subtle differences in the processing of EFEs [34]. The effect of intensity on eye scan paths was dependent on the type of expression, and will be discussed in more detail below.

Based on the stereotype and structural similarities theories, it was also predicted that different expression types would be recognized with more or less ease dependent on the sex of the face showing the expression. However, we failed to find support for these theoretically driven predictions. In fact, for the expression stimuli used in the current experiment, we observed the opposite pattern: happy expressions were more accurately identified from male faces, while disgust was more accurately identified from female faces. The calculation of Bayes factors based on previous effect sizes however suggests that the data may show evidence that is most consistent with the null hypothesis. We also observed a speed/accuracy trade-off in the recognition of male and female EFEs, with female faces being recognized with more accuracy, and male expressions recognized with more speed. In line with previous findings, we did not observe any significant differences in the pattern of eye scan paths for male and female faces. Finally, in contrast to earlier findings [58], we did not find any evidence for differences in the way women and men recognize and scan facial expressions.

Analyses of accuracy and RT revealed an influence of the sex of the face and the sex of the observer, with female faces recognized more accurately by both sexes, although this female face advantage was larger for female participants. Conversely, although not significant, response times for female expressions were faster than for male expressions. Male participants also appeared to show a relatively greater difference in RT, being more than 100ms slower to identify female compared with male expressions. However, these results only partially support the attachment promotion theory which suggests that females are more adept at EFE identification in general [48–49].

Although female participants showed some degree of superiority in correctly classifying female compared with male faces, the only evidence for a more generalized pattern of female superiority was the finding of overall faster RTs. We also found no support for the fitness to threat theory of female superiority in identifying specific threat related emotions [48–49]. Rather, the present findings suggest that perhaps any differences in emotion recognition abilities between male and female participants lie in the gender of the face that they are observing. Although these findings show some support for sex differences in the processing of male and female EFE information, similarities in eye tracking parameters are not consistent with broader, more general differences in the cognitive systems underlying EFE recognition in male and female participants. However, the exploration of gender-based differences in this paper was based on relatively small sample sizes, and these should be considered when interpreting the observed effects. Although the absence of some predicted effects may reflect low statistical power, where predicted effects were not observed, or were observed in the opposite direction, the calculation of Bayes factors (based on previous effect sizes) suggested that the current data typically showed support for the null hypothesis.

For expressions at 55% intensity, dwell times were longer on the mouth for disgusted compared with happy and surprised expressions. Consistent with this finding, it has been shown that the mouth region may reveal information for expressions of disgust that can be used with high optimality [64]. The finding of relatively increased attention to the mouth of disgust expressions at lower intensities is similar to earlier findings [64], and supports the conclusion that when judging more ambiguous or lower intensity expressions, greater attention is allocated to those regions that contain emotion specific diagnostic information [64]. However, this finding may also reflect methodological issues around the selected stimuli. The process for creating moderate intensity expressions involved morphing faces expressing emotional content with neutral expressions. As a result of this process, some faces may appear obscure, and this is particularly true for open mouthed expressions of disgust. Here, when morphed with neutral the tongue can appear translucent and may attract the focus of attention. These issues call in to question the extent to which modified intensity expressions truly resemble real world expressions to the greatest degree [33].

While the morphing process for creating modified intensity expressions is subject to certain limitations, such as those described above, this remains the most common way to create mixed intensity emotionally expressive face stimuli. A set of more naturalistic expressions rated for emotional intensity would help to overcome some of these difficulties and would be more ecologically valid. Alternatively, the use of dynamic faces showing increasing emotional intensity would better reflect task demands in the real world where expressions are seldom still. A further methodological consideration involves the predefined placement of AOIs across all faces. Although the eye and the mouth AOIs were consistent in terms of their size and shape, different facial proportions mean that there was some degree of variation in the contents of the AOIs for different faces. For example, for some faces the mouth AOI included the philtrum or Cupid’s bow, but this was absent for other faces. The predetermined placement of AOIs however limits the inherent subjectivity of manually placing AOIs for each expression.

A final issue to consider is the inclusion of a ‘neutral’ option that participants could select if the face appeared to show little or no emotional content. Even for very low intensity expressions, neutral responses were recorded as incorrect, despite being 90% neutral and only 10% expressive. However, this design allowed us to explore whether or not participants were sensitive to very low levels of emotional content, and the effects of lowered intensity on eye scan paths. Furthermore, including the neutral option also made the task more representative of task demands during real world social interactions, where faces expressing little emotional content are perhaps more likely to be dismissed as neutral.

## Conclusion

Here we show that during free viewing of EFE stimuli, accuracy rates, RTs and eye scan paths can vary with the type and degree of emotional content on show. In particular, we found that fearful and happy expressions produce the most pronounced effects, with fearful expressions recognized with the least speed and accuracy, while happy expressions were recognized with the greatest speed and accuracy. The identification of fearful and happy expressions may therefore be supported by different underlying mechanisms for emotion recognition, and this conclusion is supported by the observation of differential eye scan paths for these expressions. Although dwell time is typically greater on the eyes compared to the mouth across all expressions, this effect was particularly pronounced for fearful expressions, and was least pronounced for happy faces. The observed effects in relation to the sex of the face were generally complex, and were dependent upon both the intensity, and the emotional content, of the expression. We would suggest that future studies should consider manipulating and examining the sex of the expressive face, as well as the effects of intensity and emotion. In contrast, observer gender did not interact with any of the factors, and no differences in eye scan paths were observed between male and female participants. These findings fail to support theories of a general female superiority effect, or sex specific processing of emotional faces.

The results reported here provide a detailed account of emotion recognition in a neurotypical sample and shows that various parameters, including accuracy, RT, and dwell time on the eyes and the mouth are sensitive to differences in the type of expressions, the intensity of the expression, and the gender of the face displaying the expression. The extent to which these variables affect similar parameters in populations that are characterized by impairments in emotion recognition might help to elucidate the underlying mechanisms for these problems. For example, individuals with psychopathic tendencies [69–73], and patients with autism [74], and schizophrenia [75], show impaired EFE recognition abilities, and these impairments may reflect abnormalities in the allocation of attention for affective faces [61, 76–78]. Analyses of eye scan paths may also help to elucidate differences in the ways that these disorders manifest in male and female patients. Similarly, reduced attention to the eye region with increasing age may also explain relatively impaired emotion recognition abilities among the elderly [79].

## Supporting Information

S1 File Accuracy minimal dataset. (SAV)

S2 File RT minimal dataset. (SAV)

S3 File Dwell Time minimal dataset. (SAV)
